# Precision Medicine in Neurodegeneration with Brain Iron Accumulation (NBIA) Disorders: An Update on Emerging Treatments

**DOI:** 10.1002/mdc3.70736

**Published:** 2026-07-10

**Authors:** Susanne A. Schneider, Divyani Garg, Vassilena Iankova, Thomas Klopstock

**Affiliations:** ^1^ Neuroimaging Center Medical Hospital of the Johannes Gutenberg University Mainz Mainz Germany; ^2^ Department of Neurology All India Institute of Medical Sciences New Delhi India; ^3^ Department of Neurology München Klinik Harlaching Munich Germany; ^4^ Friedrich Baur Institute, Department of Neurology LMU University Hospital, Ludwig‐Maximilians‐University Munich Munich Germany; ^5^ Munich Cluster for Systems Neurology (SyNergy) Munich Germany; ^6^ German Center for Neurodegenerative Diseases (DZNE) Munich Germany

**Keywords:** disease‐modifying, NBIA, PKAN, PLAN, precision medicine

## Abstract

**Background:**

Neurodegeneration with Brain Iron Accumulation (NBIA) is a heterogeneous group of heritable, mostly recessive, progressive neurodegenerative diseases characterized by iron deposition in the basal ganglia and brainstem. There are no solid global epidemiological data on prevalence and incidence of NBIA subtypes, but registry data and expert opinion suggest PKAN, BPAN, PLAN, and MPAN are the most common subtypes. NBIA disorders present with a wide spectrum of clinical symptoms, including movement disorders (dystonia, parkinsonism, chorea), pyramidal involvement (eg, spasticity), speech and cognitive deficits, motor and cognitive slowing, and ocular abnormalities. Treatment remains symptomatic, though several new drugs are in development.

**Objectives and methods:**

Following our review published in 2021, this article provides an updated summary of recent developments. We discuss the rationale of new compounds, summarize clinical trials or—in their absence—preclinical studies for NBIA subtypes. The article is divided into two sections: one section on general approaches based on the shared feature of increased iron in the brain; and the second section on tailor‐made, mechanistic treatments for the various NBIA subtypes targeting the specific molecular and cellular pathways of the affected enzyme including gene therapy.

**Results and conclusions:**

In summary, randomized controlled trials in NBIA have not yet demonstrated substantial benefit, neither for iron removal, in general, which appears to be clinically ineffective in most subtypes, except aceruloplasminemia; nor for subtype‐specific approaches. Several ongoing studies are exploring more dedicated compounds in this exciting field.

Neurodegeneration with Brain Iron Accumulation (NBIA) comprises a heterogeneous group of disorders characterized by iron deposition in the basal ganglia and brainstem—particularly the globus pallidus and substantia nigra—visible on MRI. Clinically, NBIA presents with progressive extrapyramidal and pyramidal symptoms (eg, dystonia, chorea, parkinsonism and spasticity), alongsideoptic, cognitive, and psychiatric features, leading to early disability and reduced quality of life.

Multiple pathophysiological pathways are implicated, including coenzyme A biosynthesis, lipid metabolism, iron homeostasis with iron overload contributing to oxidative stress, protein misfolding, autophagy–lysosomal dysfunction, neuroinflammation, ferroptosis, and additional pathways of unknown function. Notably, only aceruloplasminemia and neuroferritinopathy result from primary defects in iron homeostasis; in other NBIA forms, iron accumulation likely represents an epiphenomenon, as seen in aging and other neurodegenerative diseases.^1^ At present, only symptomatic treatments are available, underscoring the need for disorder‐specific therapeutic disease‐modifying therapies.

This review updates our 2021 publication^2^ and summarizes recent advances in mechanistic treatment strategies. We review and discuss iron chelation as a generic approach, and disease‐specific strategies, including metabolic bypass, modulation of downstream cellular effects, small molecules, genetic therapies, and enzyme replacement.

Therapeutic development for rare disorders such as NBIA is challenged by clinical heterogeneity limited patient numbers, and the use of traditional study designs and methodologies which may prove inadequate.^3^ Collaboration among experts, early regulatory engagement, and involvement of patients and families help overcome these challenges.

## The General Approach: Prevention of Iron Build‐Up and Removing the Iron

A general mechanistic strategy for NBIA treatment is to prevent or reduce iron accumulation. Three approaches target different stages of the pathophysiological cascade: (1) correcting the underlying genetic defect via gene therapy, (2) limiting cellular iron uptake, and (3) removing iron with chelators.

Only two NBIA subtypes—aceruloplasminemia and neuroferritinopathy—result from mutations in genes directly regulating iron homeostasis. In all other NBIA forms, the link to iron metabolism is unclear, and brain iron accumulation is considered an epiphenomenon, also seen in normal aging and other neurodegenerative diseases such as Parkinson's disease and Alzheimer's disease.^4^, ^5^ Iron overload promotes oxidative stress, protein misfolding, autophagy‐lysosomal dysfunction, neuroinflammation, and ferroptosis (iron‐dependent apoptosis).

### Gene Therapy

Gene therapy is widely regarded as a promising treatment approach for a range of inherited diseases, particularly metabolic disorders. Gene therapy aims to regulate, compensate for genetic defects, deliver functional genes or modify existing gene expression in order to produce a therapeutic benefit in target tissues, eg, in neurons or glia, in genetically based conditions. In recessive diseases, it provides a healthy gene copy to restore protein function. This can be achieved by introducing functional copies of a gene to replace defective ones, disabling malfunctioning genes through transcriptional or translational modifications, or inserting a new gene that provides a therapeutic effect.^6^, ^7^

In gene therapy, the gene of interest is delivered to target cells using a vector. There are two major categories of vectors^6^, ^8^: viral and non‐viral. Non‐viral approaches include synthetic and natural polymers that take advantage of biologically derived cellular components. While they are not constrained by the size of DNA inserts and induce relatively low immunogenic responses, they show low selectivity and reduced gene‐transfer efficiency compared with viral systems. Therefore, viral vectors may be more attractive for therapeutic and clinical use, as they are highly selective within target tissues, while also being versatile enough to differentiate among cells in different stages of the growth cycle. Retroviruses, adenoviruses, lentiviruses, and adeno‐associated viruses (AAVs) may be used.

Among these, retroviruses and adenoviruses have been linked to potential immunogenicity and toxicity issues, raising important safety concerns. In contrast, AAVs are considered non‐pathogenic in humans and are therefore viewed more favorably with regard to immunological safety.^7^

A dozen AAV serotypes and more than 100 variants have been identified, allowing computationally targeting of a wide range of tissues.^9^ AAVs possess a small single‐stranded linear genome flanked by 145‐nucleotide inverted terminal repeats (ITRs), which can accommodate small therapeutic inserts or be replaced with genes of interest. For example, AAV vectors can be used to deliver therapeutic proteins, antibodies, microRNAs, or enable precise DNA insertions and deletions to modify the genomic profile of host cells.^7^

Gene therapy may be particularly advantageous for neurological disorders, including NBIA syndromes, because many vectors are capable of crossing the blood–brain barrier. When combined with cell type‐specific promoters and enhancers, this enables targeted delivery to affected brain regions.^7^

Preclinical studies have indicated that direct intravenous administration of AAV vectors may be as effective in reversing neuronopathic phenotypes as intracerebroventricular injections that selectively target the central nervous system (CNS).^7^ Furthermore, several completed and ongoing clinical trials provide cautious optimism regarding the therapeutic potential of AAV‐based strategies for neurological diseases.^7^ These studies support both the clinical safety and overall efficacy of AAV vectors, making them a promising therapeutic option for NBIA disorders. In NBIA, some programs are in late preclinical development, with PLAN the most advanced, preparing for first‐in‐human trials (see below).

### Reduction of Iron Cellular Intake

Transmembrane transferrin receptor 1 (TfR1) mediates endocytotic iron uptake from transferrin. In NBIA cell lines—including PANK2‐, C19orf12‐, and PLA2G6‐mutant fibroblasts—reduced TfR1 palmitoylation has been identified as a key mechanism contributing to brain iron accumulation.^10^

A first‐generation semisynthetic artemisinin derivative, enhances TfR1 palmitoylation, reducing iron uptake. While widely used for severe or multidrug‐resistant malaria^11^ and investigated for cancers linked to iron overload, some mechanisms may overlap with NBIA pathophysiology, potentially worsening cellular damage.^12^, ^13^ However, while experimental evidence exists for plausible use of artesunate as a repurposed medication, this has not been studied in NBIA disorders. Notably, artesunate carries neurotoxicity risks, primarily affecting brainstem (auditory and vestibular) and cerebellar function.^14^

### Iron Chelation

Iron chelators bind iron with high affinity and remove it from tissues, effectively treating systemic iron overload disorders such as transfusional hemochromatosis and thalassemia,^15^ while it may worsen Parkinson's disease.^16^ The first report of iron chelation in NBIA appeared in 1968,^17^ showing no clinical improvement, and subsequent case reports over 40 years have yielded mixed and inconclusive results.^18^

Three chelators are clinically available: deferiprone, deferasirox, and desferrioxamine (deferoxamine), differing in chemistry, administration, pharmacokinetics, and pharmacodynamics. Deferiprone, with low molecular weight, favorable lipophilicity, blood–brain barrier penetration, and oral administration, is considered the most suitable for NBIA.^19^, ^20^ Other chelators, beyond these three, have not been investigated in NBIA (for potential compounds, see^21^).

While radiologically effective, clinical benefit is often limited. Aggregated case reports suggest that iron chelation is most effective in aceruloplasminemia, particularly when initiated before neurological symptoms manifest.^22^ However, robust evidence is lacking, and there is an unmet need for RCTs. In neuroferritinopathy, a RCT in 40 patients, randomized 1:1 to deferiprone vs. placebo, is currently done at the University of Cambridge, UK.^23^ In PKAN, clinical benefits are limited; a randomized trial suggested modest effects in patients with milder, later‐onset disease.^24^ For other NBIA subtypes, chelation is currently not recommended due to lack of evidence and potential risks.

In the future, development of more refined pharmacological strategies for targeting iron including iron‐mobilizing agents, selective chelators targeting specific cellular iron pools, chelators with reduced iron‐binding affinity, chelators that are selective for Fe^2+^, and compounds that block ferroptosis without directly chelating iron will provide further insights.^5^

## Disease‐Specific Strategies Based on Molecular Mechanisms of the NBIA Subtypes

There are concerted efforts to translate insights into the pathophysiology for the sake of future disease modifying treatments (Table 1). In NBIA disorders, most work has been done for PKAN, where the gene was the first to be discovered. The following section reviews disease‐specific advances, beginning with the more prevalent subtypes. Notably, reported frequencies vary substantially across registries, and robust global epidemiological data remain unavailable. Population‐genetic estimates of lifetime risk for recessive NBIA forms revealed pooled risks of 0.88 per 100,000 based on the global gnomAD dataset and 0.92 per 100,000 in the European gnomAD dataset—values that substantially exceed previously reported population‐based estimates.^25^

**TABLE 1:** Summary of treatments in neurodegeneration with brain iron accumulation (NBIA)

Therapy	Mechanism of Action	Development Stage (as of 2025)	NBIA Subtype	Comments
Gene Therapy (AAV‐based)	Gene replacement to restore protein expression	Early preclinical (PKAN), late preclinical with dose‐finding (PLAN)	PLAN, PKAN	Application in PLAN most likely includes dose, targeting, vector regulation
Iron Chelation—Deferiprone	Chelates Fe^3+^ and crosses BBB → reduces brain iron	Phase II–III completed	PKAN, aceruloplasminemia, neuroferritinopathy	Radiological effect > clinical effect; best in atypical PKAN and aceruloplasminemia
Iron Chelation—Deferasirox / Desferrioxamine	Iron chelation	Not trialed systematically in NBIA	Aceruloplasminemia, neuroferritinopathy	Limited BBB penetration; variable results in small case series
Coenzyme A Supplementation	Direct CoA replacement	Preclinical	PKAN, CoPAN	Unstable in serum; poor membrane permeability
Pantothenate (Vitamin B5)	Increases substrate availability for PANK2	Anecdotal data only	PKAN (atypical)	Helps only with residual enzyme activity; no effect in classic PKAN
Fosmetpantotenate (RE‐024)	Membrane‐permeable phosphopantothenate precursor	Phase III completed (negative)	PKAN	Development discontinued after trial failure
4′‐Phosphopantetheine (CoA‐Z)	Bypasses PANK2 defect → restored CoA synthesis	Ongoing clinical trials (Phase II/III)	PKAN	Only alternative substrate still clinically active
Pantethine	Precursor to pantetheine → 4′‐phosphopantetheine generation	Open‐label pilot completed	PKAN	Serum instability of pantetheine; no clear efficacy
Acetyl‐4′‐phosphopantetheine / Cyclic Phosphopantothenic Acid	Stable precursors for CoA biosynthesis	Preclinical	PKAN	No human data
PANK Activators—Pantazines (PZ‐2891)	Activate cytosolic PANK isoenzymes (esp. PANK3)	Preclinical; halted	PKAN	Poor pharmacokinetics
PANK Activators—Pyridazines (BBP‐671)	Allosteric PANK activators	Early clinical attempt terminated	PKAN	Highly variable plasma levels; discontinuation
PANK Activators—VTAC Compounds	Brain‐penetrant PANK3 activators	Preclinical; IND‐enabling stage	PKAN	Phase 1/2 trial planned
Rapamycin / mTORC1 Inhibition	Enhances autophagy	Preclinical	BPAN	Non‐specific; unknown long‐term safety in children
Cardiac Glycosides (Autophagy Restorers)	Restore autophagic flux in WDR45‐deficient neurons	Preclinical	BPAN	Toxicity concerns; early stage
SNAP29 O‐GlcNAcylation Inhibitors	Improve autophagosome–lysosome fusion	Preclinical	BPAN	Mechanistic but early data only
D‐PUFAs (RT001)	Reduce lipid peroxidation; stabilize membranes	Phase II/III (negative for primary endpoint)	PLAN, INAD	Development discontinued; mixed case reports
Desipramine	Inhibits acid sphingomyelinase → reduces ceramides	Very early (n = 4 pilot)	PLAN	No published results; safety concerns
GLP‐1 Receptor Agonists (Semaglutide)	Anti‐inflammatory; reduces apoptosis/necroptosis	Preclinical	PLAN	Not recommended clinically; pediatric safety considerations
Pharmacological Chaperones for PLA2G6	Stabilize misfolded mutant enzyme	Preclinical	PLAN	Target identification ongoing
C19orf12 Overexpression / Modulators	Restores MAM function, reduces ER stress	Preclinical (flies)	MPAN	No mammalian model yet
Ceruloplasmin Replacement Therapy	Restores ferroxidase activity	Preclinical (mouse)	Aceruloplasminemia	Improves motor and iron biology; no human data
Gene Therapy for Ceruloplasmin	Gene supplementation	Conceptual only	Aceruloplasminemia	Not yet initiated
Iron Chelation in Neuroferritinopathy	Removal of excess iron	DEFINE Trial (ongoing, double‐blind RCT)	Neuroferritinopathy	Mixed previous case results; 7 T MRI biomarker study

Abbreviations: AAV, Adeno‐Associated Virus; BBB, Blood–Brain Barrier; CoA, Coenzyme A; CoPAN, COASY Protein‐Associated Neurodegeneration; Fe, iron; GLP‐1, Glucagon‐Like Peptide‐1; NBIA, Neurodegeneration with Brain Iron Accumulation; PANK2, Pantothenate Kinase 2; PKAN, Pantothenate Kinase‐Associated Neurodegeneration; PLAN, PLA2G6‐Associated Neurodegeneration; RCT, randomized controlled trial; T, Tesla; VTAC, Virtus Therapeutics Activating Compounds; WDR, WD Repeat Domain.

### BPAN

BPAN clinically presents with childhood‐onset seizures, developmental delay, loss of expressive language, stereotypies, behavioral abnormalities, and intellectual disability. In adulthood, movement disorders such as dystonia and parkinsonism typically emerge. To date, there is no well‐publicized industry clinical pipeline for BPAN yet. Treatment remains symptomatic, management guidelines have been established.^26^

Pathologically, BPAN is a tauopathy, with neurofibrillary tangles, pre‐tangles, and neuropil threads containing 3‐R and 4‐R tau isoforms, along with nigral neuronal loss, gliosis, and iron accumulation.^27^ Alpha‐synuclein deposits are absent.

BPAN results from de novo mutations in the X‐linked *WDR45* gene, which encodes WIPI4, a WD40 repeat‐containing protein involved in early autophagy.^28^, ^29^ Notably, variants in this gene are also linked to other neurodegenerative phenotypes, including Rett‐like syndrome, intellectual disability, epileptic encephalopathies and potentially also specific malignancies.^29^ WIPI4 is one of four mammalian homologs of yeast Atg18. BPAN patient‐derived lymphoblastoid cells show reduced autophagy and accumulation of ATG9A‐positive structures, indicating impaired autophagosome formation.^30^ A link to ferroptosis has been proposed.^31^ Knockout mouse models of BPAN^32^, ^33^ show poor motor coordination, severe learning and memory deficits, and extensive axonal swelling with spheroids.

Therapeutic, currently preclinical approaches focus on autophagy activation and ER stress inhibition WDR45 appears to function as a critical regulator of stress granule dynamics not only in BPAN, but also in amyotrophic lateral sclerosis implying broader relevance of WDR45 to neurodegenerative diseases in general.^34^

#### Activation of Autophagy

Mechanistic Target of Rapamycin (mTOR) is a kinase that inhibits autophagy by phosphorylating Ulk1, essential for autophagy initiation.^35^ mTOR also regulates cell growth, lipid and nucleotide synthesis, organelle biogenesis, and adaptation to feeding and fasting, responding to nutrients, growth factors, and stressors.^36^ Rapamycin inhibits mTOR, thereby activating autophagy. It is used clinically as an immunosuppressant and is under investigation for cancer and neurodegenerative diseases, showing neuroprotective effects in models of Parkinson's disease and Alzheimer's disease via enhanced autophagic clearance.^37^, ^38^, ^39^

In a BPAN mouse model, rapamycin reduced ER stress, partially restored autophagy, and decreased neuronal death, though ER expansion remained unchanged.^33^ While mTOR inhibition is being tested in genetic Parkinson's disease,^39^ no clinical trials in BPAN are known.

More recently, cardiac glycosides that restore autophagy in BPAN cell lines were discovered in a drug screen.^40^ Activating autophagosome‐lysosome fusion, eg, via inhibiting O‐GlcNAcylation of SNAP‐29, may represent another therapeutic strategy in BPAN.^41^

#### Gene Therapy for BPAN

Proof‐of‐concept gene therapy studies (also see PLAN section) have been conducted in mouse models which resulted in successful expression of human WDR45 transcripts and WIPI4 protein in the brain tissue, rescue of hyperactive behavior, and correction of autophagy markers.^42^

In summary, research on BPAN remains in early stages, with no clinical trials to date. Preclinical studies suggest mTORC1 inhibitors like rapamycin may be beneficial, though likely non‐specific. Enhancing autophagy is a key proposed mechanism to counteract neurodegeneration and could apply to other disorders. Anti‐tau strategies, based on Alzheimer's research, have not yet been tested for BPAN.

### PKAN

PKAN, the best‐studied NBIA subtype, is caused by mutations in *PANK2* on chromosome 20, which encodes the mitochondrial enzyme pantothenate kinase 2. This enzyme catalyzes the first step of CoA biosynthesis, phosphorylating pantothenate to 4′‐phosphopantothenate. CoA is essential for the citric acid cycle, fatty acid oxidation, amino acid metabolism, and neurotransmitter synthesis.

Two clinical PKAN phenotypes are recognized: classic and atypical.^43^ Dystonia is a key feature, often affecting the lower limbs, with orolingual involvement helping distinguish it from other dystonias (eg, DYT1). The atypical form has later onset, slower progression, and a milder course.

Due to its relative frequency, PKAN has been the primary focus of clinical trials and research on disease‐modifying therapies in cell, animal, and human studies.

### Disease Modifying Approaches for PKAN and CoPAN

#### *PANK2* Gene Therapy

Gene therapy for PKAN using AAV9 vectors carrying the full‐length human *PANK2* gene is in early development.^44^ Preclinical studies involve stereotactic injections into the globus pallidus in mice. No results have been reported, as the project remains at an early stage.

#### Iron Chelation

Over the past decades, case reports of chelation therapy in PKAN led to structured clinical trials. A phase II trial in nine PKAN patients treated with deferiprone showed radiological improvements but no clinical benefit.^45^ Subsequently, six NBIA patients (five PKAN, one idiopathic) treated for 36–48 months had mixed outcomes, with radiological improvement but clinical worsening or stabilization.^46^ The largest study—a placebo‐controlled, double‐blind, multicenter trial with 78 patients—showed reduced basal ganglia iron on MRI after 18 months (plus an 18‐month extension), yet no significant clinical benefit, only a non‐significant trend toward slower progression. This discrepancy likely reflects irreversible neuronal damage or suggests that iron accumulation is a secondary bystander rather than a primary driver.^24^ Overall, chelation therapy has limited clinical effect but may benefit subsets of patients, such as those with residual enzyme function or atypical PKAN.

#### Enhancing Coa Biosynthesis Using Alternative Substrates

A targeted therapeutic approach for PKAN is the use of alternative substrates to bypass the PANK2 defect and restore CoA biosynthesis. Both intermediates and direct CoA supplementation have been tested. Among these, 4′‐phosphopantetheine (CoA‐Z, see below) shows the greatest promise and is currently in clinical trials, while development of other compounds was halted due to serum instability or lack of clinical efficacy.

##### Coenzyme A–The Direct Supplementation Approach

CoA supplementation showed multiple benefits in preclinical NBIA models. In human‐derived pluripotent stem cells differentiated into PKAN neurons, CoA improved neuronal viability, excitability, and firing activity, reduced ROS, and restored mitochondrial function.^47^ It also rescued phenotypes caused by CoA depletion in cultured cells, *C. elegans*, and Drosophila models,^48^ and partially restored cell counts in PANK‐depleted Drosophila, though it was less effective and more toxic than pantethine.^49^ CoA supplementation also increased TfR1 palmitoylation in fibroblasts with *PANK2* mutations.^10^

However, CoA is unstable—90% degrades within 30 min in serum‐containing medium—and cannot cross cell membranes, being rapidly converted to its precursor, 4′‐phosphopantetheine, which is stable, bioavailable, and membrane‐permeable.^48^

In CoA synthase (COASY) protein‐associated neurodegeneration (CoPAN), caused by *COASY* mutations, CoA supplementation failed to improve phenotypes in flies and cells.^48^ This is particularly limiting, as COASY is required to convert the precursor into CoA intracellularly.

Thus, exogenous CoA is unstable and converted to its precursor, making it ineffective for CoPAN. For PKAN, where CoA supplementation may rescue phenotypes, its instability raises the question of whether direct 4′‐phosphopantetheine therapy (Section II.1.d) is a superior approach.

##### Pantothenate—To Increase the Physiological Substrate

Pantothenate, the substrate for PANK2, reduces enzyme activity lowers CoA synthesis. Fibroblasts from patients with *PANK2* mutations exhibit altered morphology, iron and lipofuscin accumulation, oxidative stress, and mitochondrial lipid peroxidation.^49^ In cell experiments, pantothenate corrected abnormalities and prevented cell death only in lines with residual PANK2 activity, consistent with PKAN Drosophila and mouse studies, where it was ineffective in the absence of PANK2.^49^, ^50^

High‐dose pantothenate may benefit PKAN patients with residual enzyme activity, though controlled data are lacking. No effect was observed in a HARP patient,^51^ and in our experience, supplementation shows no meaningful clinical benefit. Because some adults with atypical PKAN perceive benefit, the current consensus guidelines recommend consideration of providing high dose pantothenate for at least 3 months, in all PKAN patients, starting at 250 mg orally, increasing weekly by 500 mg until a daily dose of 2–5 g or side effects occur.^52^ Most classic PKAN patients do not respond and discontinuation is recommended.^52^

##### Fosmetpantotenate—An Alternative Substrate

PANK2 converts pantothenate to 4′‐phosphopantothenate, but direct supplementation failed due to poor membrane permeability.^53^ Fosmetpantotenate (RE‐024, Retrophin, Inc.) was developed to cross the blood–brain barrier and neuronal membranes.^54^ Intracellularly, it is metabolized to 4′‐phosphopantothenate, providing a substrate for CoA biosynthesis and bypassing the *PANK2* defect.

Some clinical improvement was reported in an atypical PKAN patient (2.64 mg/kg/day for 12 months)^55^ and in two PKAN siblings (3 mg/kg/day for 47 weeks). However, a subsequent randomized, double‐blind, placebo‐controlled phase III trial in 84 patients failed to meet primary (PKAN‐ADL) or secondary (UPDRS part 3) endpoints after 24 weeks.^56^ The open‐label extension was terminated, and development of the drug was discontinued.^57^

##### 4′‐Phosphopantetheine—Another Alternative Substrate

4′‐Phosphopantetheine bypasses the PANK2 defect and is converted by COASY into 4′‐dephospho‐CoA and CoA. In PANK2 cell and mouse models, it showed beneficial effects.^50^ In PKAN fibroblasts, 24‐h treatment restored COASY and Tfrc expression. In mice, 14‐day treatment normalized COASY, iron homeostasis genes (Tfrc, Ireb2, Drd1), dopamine markers, and restored pyruvate dehydrogenase and complex I activity. However, as PANK2 mice display only mild late‐onset retinopathy without neurological symptoms, effects on neurological features remain unclear.

CoA‐Z, containing 4′‐phosphopantetheine, was tested in a first‐in‐human study to assess safety, tolerability, and COASY mRNA expression.^58^ Six PKAN patients (three classic, three atypical) received 14‐day treatment followed by 14‐day washout. No adverse events occurred, and COASY mRNA increased in all patients.

CoA‐Z is currently being investigated in three trials: a randomized, double‐blind, placebo‐controlled study (NCT04182763) in 60 patients with high, medium, or low doses, including a 30‐month open‐label extension at the medium dose, showing safety, tolerability, and dose‐dependent biomarker response^58^; a Netherlands‐based open‐label, within‐subject dose‐escalation study in 10 patients (NL‐OMON550013), recently completed, with results pending; and a placebo‐controlled, double‐blind trial at UCL in 24 patients (ISRCTN10664670), with an 18‐month open‐label extension, currently ongoing.

##### Pantethine–Another Alternative Substrate

Pantethine, a derivative of pantothenic acid, is enzymatically converted into two pantetheine molecules, which can be phosphorylated to 4′‐phosphopantetheine, a substrate for CoA biosynthesis. In *PANK2* knockout models, pantethine rescued clinical phenotypes in mice^59^ and restored CoA levels and mitochondrial function in Drosophila.^49^ Serum instability of pantetheine due to pantetheinase activity has been noted as a potential limitation.^60^

Pantethine is used as a nutritional supplement for hyperlipidemia. In a single‐arm, open‐label trial of 15 PKAN patients, 24 weeks of treatment at 60 mg/kg/day did not alter CoA levels or improve motor symptoms, though a possible slowing of motor decline was suggested.^61^

#### Other Alternative Substrates

Other compounds in development include S‐acetyl‐4′‐phosphopantetheine, an acylated form of 4′‐phosphopantetheine, and cyclic phosphopantothenic acid. In preclinical studies, both were serum‐stable and capable of rescuing PANK‐deficiency phenotypes.^60^ No patient studies have been conducted.

### Enhancing CoA Biosynthesis by *Activation of Isoenzymes*

PANK activators can stimulate multiple enzyme isoforms, so enhancing PANK1 and PANK3 activity may compensate for PANK2 deficiency. Recent screening of large compound libraries has identified three classes of PANK3 activators: pantazines, pyridazines, and VTACs.

#### Pantazines and Pyridazines

Pantazine compounds, specifically PZ‐2891, stabilize the active form of PANK3, making it resistant to acetyl‐CoA inhibition.^62^ In preclinical studies, PZ‐2891 increased CoA levels in cultured cells and in a mouse model, raising liver and brain CoA, improving weight, locomotor activity, and extending survival from 52 to 150 days. However, unfavorable pharmacological properties have prevented trials in NBIA patients.

Next‐generation pyridazine PANK activators were developed with better pharmacokinetics, including enhanced brain penetration, stability, and higher PANK2 affinity.^63^ In early clinical trials, BBP‐671—originally discovered at St. Jude's and further developed by CoA Therapeutics/BridgeBio—showed highly variable blood concentrations, with some exceeding safety limits. The cause of these variations was unclear, leading to discontinuation of the program.^64^

#### Virtus Therapeutics Activating Compounds (VTACs)

VTAC1–14, a series of PANK3 activators, demonstrated favorable pharmacokinetics and no apparent toxicity in preclinical mouse studies.^65^ Early candidates, VTAC1 and VTAC2, are being further evaluated for PKAN‐relevant effects, including CoA pathway activation, mitochondrial metabolism, and iron homeostasis, aiming to rescue PANK‐related phenotypes. A Phase 1/2 trial is planned (oral communication, Ben Mamoun, October 2025).

In summary, clinical research to find disease‐modifying treatment approaches for PKAN has been quite active in the last decade. The research centers on two main strategies—(1) Prevention or removal of brain iron accumulation and (2) bypassing or compensating for the PANK2 enzyme defect by the use of alternative substrates or activation of isoenzymes. Iron chelation has only limited clinical benefits but may be useful in a subset of patients such as those with residual enzyme function who clinically present with atypical PKAN. Trials exploring alternative substrates were unsuccessful, leaving 4′‐phosphopantetheine as the only compound in clinical trial. Likewise, PANK3 activators were unsuccessful in initial trials; VTACs are currently in the preclinical stage of development. PANK2 gene therapy is also in its early stages.

### PLAN

Mutations in *PLA2G6* on chromosome 22 cause PLAN by disrupting the intracellular, calcium‐independent phospholipase A2 group VI, which hydrolyzes the sn2 ester bond in glycerophospholipids, releasing polyunsaturated fatty acids and lysophospholipids.^66^ Preclinical studies show altered phospholipid metabolism with ceramide accumulation, lysosome expansion and mitochondrial inner membrane damage.^67^ ^.^ ^68^ In iPLA2‐VIA‐deficient Drosophila, shortened phospholipid acyl chains induced ER stress and promoted α‐synuclein aggregation.^69^ INAD mouse models revealed abnormal Purkinje cells in line with prominent cerebellar features seen in patients.^68^

PLAN encompasses several phenotypes: infantile neuroaxonal dystrophy (INAD), atypical neuroaxonal dystrophy (ANAD), and dystonia‐parkinsonism.^70^ Neuroaxonal dystrophy is defined by axonal spheroids observed in INAD and ANAD biopsies.^71^ INAD typically manifests in the first year of life, with 67% of patients showing developmental delay and 37% never walk independently.^72^ Progressive psychomotor regression is universal, alongside ataxia, early truncal hypotonia progressing to spastic tetraparesis, neuro‐ophthalmologic abnormalities, and loss of ambulation within 5 years. Cerebellar atrophy is the most common imaging finding, with 48% showing brain iron accumulation.^73^, ^74^

ANAD presents later (median onset ~4 years) and progresses more slowly, with ataxia, gait impairment, dystonia, dysarthria, and spastic paresis. A subset of PLAN patients develops early‐onset parkinsonism (<40 years) with dystonia, gait and cognitive abnormalities (dystonia‐parkinsonism, PARK14). Brain iron accumulation may occur but is not universal, so not all PLAN cases strictly meet NBIA criteria.

Therapeutic development has focused primarily on INAD. Below, we summarize recent advances in drug development.

#### Gene Replacement Therapy

Adeno‐associated viruses (AAVs) can deliver genes for therapeutic purposes. For PLAN, Bloomsbury Genetic Therapies (UCL), Andelyn Biosciences (partnered with INADcure) and others^68^ are developing gene therapy based on successes in related disorders^75^ and PLA2G6‐INAD animal models.^76^ Untreated PLA2G6‐INAD mice show early motor incoordination, muscle atrophy, gait impairment, and reduced lifespan. A single presymptomatic dose of AAV9.hSyn1.hPLA2G6 improved weight, prevented motor decline, extended lifespan, and enhanced neuronal viability while reducing neurodegeneration. However, neuronal counts remained below wild‐type levels, and neuroinflammation persisted. Dose‐finding studies are ongoing to prepare for first‐in‐human trials. Notably, gene therapy for large genes such as *PLA2G6* poses substantial challenges, driven by both gene size and the need for stringent control of expression. Studies are also needed to determine the best location to deliver gene therapy in NBIA.

#### Iron Chelation in PLAN

In PLAN, treatment with deferiprone (at a dose of 30 mg/kg/d) led to increase of markers of iron metabolism such as transferrin, haptoglobin and hemopexin. However, clinical results were not reported^77^ and iron chelation is explicitly not recommended in the current PLAN consensus guidelines.^78^

#### Deuterated Polyunsaturated Fatty Acids (D‐PUFAs)—Slowing Down Lipid Peroxidation

The brain is rich in polyunsaturated fatty acids (PUFAs), which are key components of lipid membranes and help reduce oxidative stress by limiting lipid peroxides and reactive oxygen species (ROS) accumulation.^79^, ^80^ Deuterated PUFAs (D‐PUFAs) have been shown to restore mitochondrial membrane potential in cultured fibroblasts and partially improve locomotor function in a Drosophila *PLA2G6* knock‐out model.^67^

D‐PUFAs are under investigation for several neurodegenerative and neuromuscular disorders, including Friedreich's ataxia, late‐onset Tay‐Sachs, and progressive supranuclear palsy. In a recent phase I/II double‐blind, placebo‐controlled trial in Friedreich's ataxia, RT001 was well tolerated, with diarrhea as the only treatment‐related adverse event.^81^

In NBIA, D‐PUFAs (RT001) have shown mixed results: a 2 year‐old INAD patient improved on 3.6 g/day RT001, particularly in bulbar and ocular functions, and achieved disease stabilization after one year.^82^, ^83^ A 5‐year‐old patient initially showed gain in fine motor skills, attention, and social interaction, but this was not sustained, and treatment stopped after six months.^82^

A subsequent prospective, single‐arm, open‐label study across two U.S. centers treated 19 INAD patients with 3.84 g/day RT001 for 12 months^84^ (Clinical Trials No: NCT03570931). Treated patients showed improved spasticity (~6.4 points improvement on the Modified Ashworth Scale), lower morbidity (82% reduction) and mortality risk (89% relative reduction) compared to natural history data. However, the primary endpoint was not met statistically, leading Retrotope/BioJiva to discontinue development. Following the company's bankruptcy, assets were acquired by RTMFP Enterprises Inc. in 2022, with no subsequent public data available.^85^

#### Desipramine—Reduction of Ceramide Accumulation

Preclinical studies in INAD flies and INAD patient‐derived neural progenitor cells suggested that desipramine may alleviate neurodegenerative phenotypes.^68^

A Duke University trial assessing off‐label desipramine in four INAD patients was completed in August 2019,^86^ but results are not yet available. Current guidelines do not recommend its use due to safety concerns and limited evidence. Desipramine inhibits acidic sphingomyelinase, which converts ceramide phosphoethanolamines and sphingomyelin to ceramide.^87^ In a PLAN loss‐of‐function fly model, desipramine reduced ceramide levels, alleviated lysosomal stress, and suppressed neurodegeneration.

#### GLP1 Receptor Agonists

High‐dose weekly administration of the antidiabetic drug semaglutide improved locomotor function and lifespan in juvenile *P* *LA2G6* ^−/−^ mice, reducing apoptotic and necroptotic mediators, neuronal loss, and neuroinflammation.^88^ Although GLP‐1 agonists are used in diabetic children similarly to adults,^89^ no trials exist in NBIA, and their use in PLAN is not recommended due to limited data and potential risks.^78^

#### Pharmacological Chaperones—Stabilizing Protein Folding of Mutant PLA2G6 Proteins

Preclinical efforts are underway to develop therapies that enhance mutant PLA2G6 function,^90^ focusing on identifying pharmacological chaperones that stabilize protein folding. Screens of compound libraries are planned. Among others, ambroxol, a pharmacological chaperone and lysosomal modulator was identified to alleviate neurodegenerative phenotypes in INAD flies and INAD patient‐derived neural progenitor cells in a drug screen.^68^

#### Overexpression of C19orf12

A potential link between PLAN and MPAN was suggested when neuronal overexpression of the MPAN‐associated C19orf12 homolog CG3740 improved disease markers in an iPLA2‐VIA‐deficient Drosophila model.^69^ C19orf12 overexpression rescued motor deficits, reduced bang‐sensitivity, enhanced dopaminergic neuron survival, suppressed brain vacuolation, normalized phospholipid acyl‐chain length, and alleviated ER stress and α‐synuclein aggregation. There is no clinical application of this yet.

In summary, for PLAN, two gene therapy programs are in the pipeline. Iron chelation therapy is not recommended. Further downstream disease‐specific approaches have failed so far to show effectiveness, including D‐PUFAs.

### MPAN

MPAN is a monogenic NBIA caused by mutations in *C19orf12* on chromosome 19. Clinically, it presents with pyramidal and extrapyramidal symptoms (eg, spasticity, dystonia), cognitive and psychiatric abnormalities, and neuro‐ophthalmologic involvement.^91^

*C19orf12* encodes a mitochondrial membrane protein localized to mitochondria, the ER, and mitochondria–ER contact sites (MAMs).^92^ MAMs regulate calcium, lipid transfer, mitochondrial fission, and autophagosome assembly, and have been implicated in other neurodegenerative diseases, including Alzheimer's disease.^93^, ^94^ In MPAN, mislocalized C19orf12 leads to elevated mitochondrial Ca^2+^ and increased oxidative stress, as shown in patient fibroblasts.^92^ Although its precise function is unknown, C19orf12 likely contributes to autophagy of defective mitochondria.

Animal models have been developed to study disease mechanisms. In Drosophila, downregulation of both C19orf12 orthologs (CG3740 and CG11671) recapitulates key features of MPAN, including climbing deficits, reduced survival, and brain vacuolation.^95^ Preclinical studies, including therapeutic testing in flies and patient‐derived cell lines, are ongoing.

In summary, the development of disease‐modifying treatment strategies for MPAN is in very early stages. There is no well‐publicized industry clinical pipeline as yet. Interesting preclinical models have enabled greater insight into the function of the C19orf12 protein and its importance for mitochondria‐ER associated membranes.

### Aceruloplasminemia

Aceruloplasminemia, due to mutations in the *ceruloplasmin* (*CP*) gene on chromosome 3, is characterized by iron accumulation not only in the brain, but also in other organs including liver and pancreas. Key symptoms are diabetes mellitus, retinal degeneration, anemia, and neurological symptoms such as cerebellar ataxia, movement disorders and behavioral changes.

The encoded protein, ceruloplasmin, is a metalloprotein with copper‐dependent ferroxidase activity which catalyzes the oxidation of Fe^2+^ to Fe^3+^. Ceruloplasmin carries the large majority of the total copper in plasma. It also enables transportation of iron in plasma.

#### Iron Chelation in Aceruloplasminemia

Iron chelation has shown promising results. A recent review summarized 48 patients reported in case studies or small series, treated with 13 different chelation strategies, mostly as monotherapy.^22^ Half of the patients (n = 24) had neurological symptoms, while the other 24 were pre‐symptomatic for neurological signs, presenting only non‐neurological features of aceruloplasminemia. Remarkably, treatment stabilized the neurological state in 50% in manifest patients and could delay the neurological onset by 10 years (median age 61 years vs. 51 years, *p* = 0.001) in the pre‐neuro‐symptomatic group. Other authors had found variable efficacy on neurological symptoms,^18^, ^96^ and some uncertain benefits on glucose metabolism and retinopathy. A randomized clinical trial is needed to clarify effects.

#### Disease‐Specific Approaches for Aceruloplasminemia

A disease‐specific approach could be enzyme replacement therapy.^97^ In *ceruloplasmin*‐knockout mice, intraperitoneal ceruloplasmin improved motor coordination, fully restored brain ferroxidase activity, rescued Purkinje cells, and reduced iron levels in the brain and choroid plexus, though liver iron remained unchanged.^98^ No data are available for patient use. Gene therapy is under preclinical investigation.

In summary, while disease‐specific treatments are under development, iron chelation appears to be effective in aceuroplasminema.

### Neuroferritinopathy

Neuroferritinopathy is an autosomal dominant, fully penetrant NBIA subtype caused by mutations in the *FTL1* (*ferritin light chain*) gene. Clinically, it presents in adulthood with progressive movement disorders—typically chorea, dystonia, and parkinsonism—with cognitive and psychiatric features. Work‐up reveals low serum ferritin, despite normal hemoglobin and serum iron levels, and characteristic MRI findings with T2 hypointensity and cavitation in the basal ganglia (especially globus pallidus and putamen) due to iron deposition.^99^

Therapeutic phlebotomy has no clinical benefit.^100^

#### Iron Chelation in Neuroferritinopathy

Data on the effect of iron chelation treatment is limited to small case series. Chinnery et al. reported three patients treated with intravenous desferrioxamine (4000 mg weekly subcutaneously for up to 14 months), or oral deferiprone (2 g t.i.d for 2 month). Two patients had no benefit and one deteriorated significantly after deferiprone treatment.^101^ Likewise, other patients failed to show improvement.^102^ On the contrary, all four patients treated by Marchand et al. improved with particular benefit in those treated early in the disease course.^103^ These findings led to initiation of the DEFINE Trial (ISRCTN15571700), a randomized, double blind, placebo controlled trial in 48 patients to assess safety and efficacy of iron chelation in neuroferritinopathy. By the time of submission of this article, about half of the patients were recruited and more patients are welcome to participate. Primary outcome will be brain iron deposition measured with 7 Tesla MRI imaging.^104^

In summary, iron chelation produced mixed results in neuroferritinopathy. The ongoing DEFINE trial will provide further insight.

## Discussion

**Figure 1:**
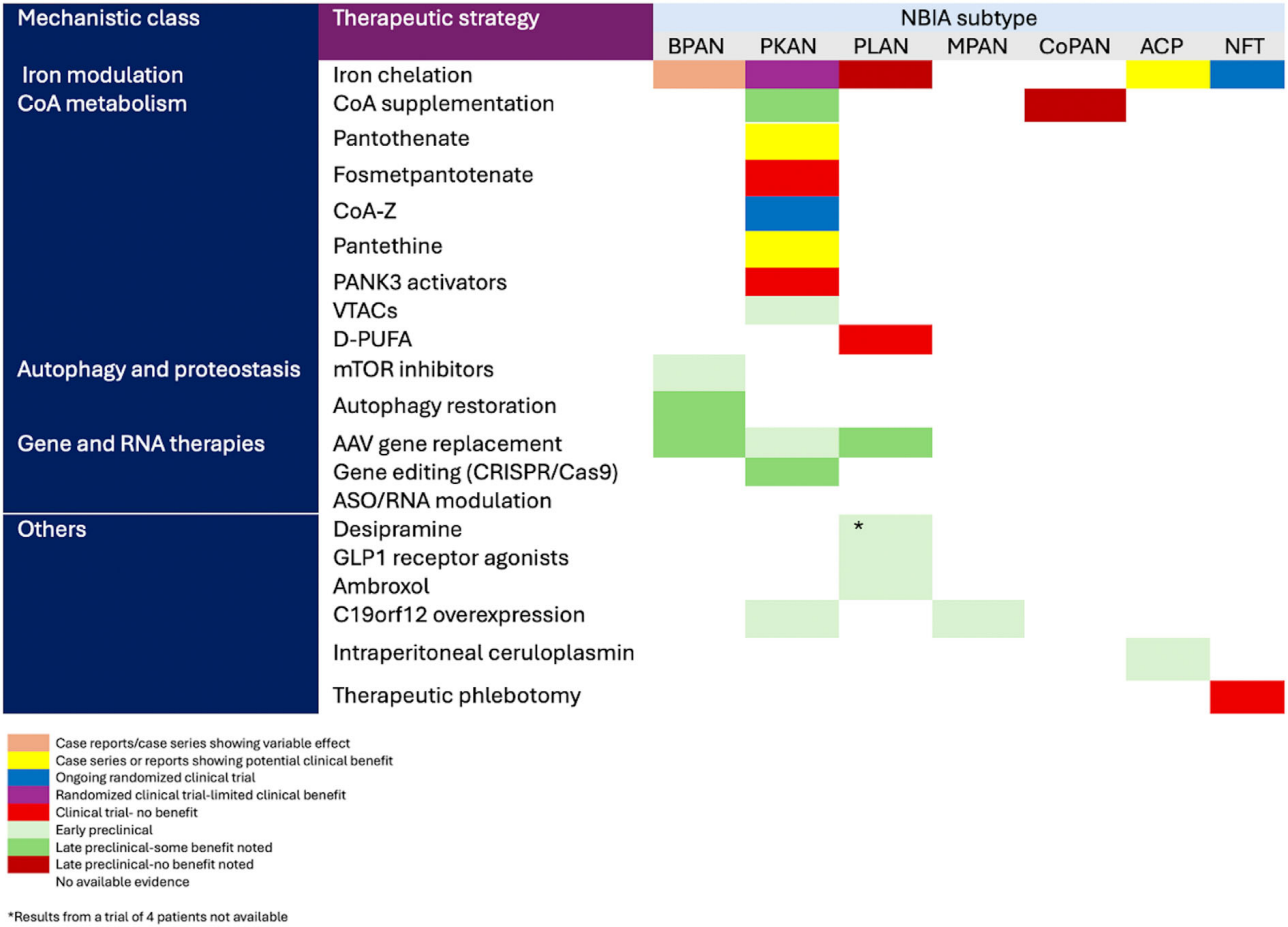
Summary of emerging disease‐modifying therapies for Neurodegeneration with Brain Iron Accumulation (NBIA) syndromes.

With growing insights into rare genetic disorders, precision medicine is becoming increasingly feasible. This review summarizes emerging disease‐modifying therapies for NBIA disorders (Fig. 1, Table 1). A major challenge in developing disease‐specific treatments remains the limited understanding of disease pathogenesis. Over the past decade, basic research has provided important insights into several NBIA disorders, enabling new therapeutic strategies, although further clarification of gene product function is still needed. Progress has also been hampered by the lack of suitable animal models: while mouse models exist for PKAN, PLAN, and BPAN; MPAN research currently relies on fly models.

As outlined here, NBIA treatment now extends beyond symptomatic iron removal to include metabolic bypass strategies, activation of isoenzymes, gene overexpression, and induction of autophagy. At present, one clinical study is actively recruiting patients with PKAN to test 4′‐phosphopantetheine‐containing CoA‐Z, while other compounds remain in preclinical development.

It will be interesting to see what other approaches will emerge from the better insight into the pathophysiology of NBIA subtypes. For example, our field has recently seen exciting new therapies for related inherited rare disorders such as spinal muscular atrophy (SMA) and Huntington's disease (HD) using antisense oligonucleotides (ASOs).^105^, ^106^ Of these, work in SMA patients have highlighted the role of ASOs in restoring protein expression by modulating splicing.^105^, ^107^ This may be helpful in disorders where the majority of genetic defects are splice site mutations. For the moment, however, this does not apply in most NBIA cases, although splice site mutations have been described in individual cases. In HD, ASOs act by reducing the toxic gain of function caused by the autosomal dominant mutation. Again, while this approach may not be feasible for the autosomal recessively inherited NBIA disorders, it may be an option for autosomal dominant NBIA subtypes (eg, neuroferritinopathy) where a healthy allele of the specific gene is present. Currently, a feasible approach for genetic therapy in NBIA disorders seems to be AAV‐assisted gene supplementation, although it needs to be demonstrated that whole brain disorders like NBIA disorders can be adequately treated with gene therapy.

Until disease‐modifying, mechanism‐based therapies become available, strategies targeting iron dysregulation may continue to be explored, but with caution. Although iron chelators can reduce brain iron accumulation, evidence for meaningful clinical benefit is limited and appears confined to specific NBIA subgroups, such as atypical PKAN, precluding their broad applicability. Induction of TfR1 palmitoylation is another mechanistically interesting approach, but its therapeutic relevance remains speculative and requires rigorous validation.

Given the neurodegenerative nature of NBIA, early patient recruitment—before irreversible neuronal damage occurs—is crucial. Advances in genetic diagnostics now allow earlier diagnosis and identification of presymptomatic mutation carriers, enabling risk‐based screening (eg, among relatives) and potentially opening a window for preventive interventions. Trial designs tailored to small cohorts may further maximize the information gained from limited patient populations.^108^

## Author Roles

(1) Research project: A. Conception, B. Organization, C. Execution; (2) Statistical Analysis: A. Design, B. Execution, C. Review and Critique; (3) Manuscript Preparation: A. Writing of the first draft, B. Review and Critique;

S.A.S.: 1A, 2B, 3B, 3A.

D.G.: 1A, 3B.

V.I.: 1B, 1C, 3A.

T.K.: 3B.

## Disclosures

**Ethical Compliance Statement:** No approval from an institutional review board or ethics committee was required for this study, as it is based exclusively on previously published literature and did not involve any new studies with human participants or animals. Informed patient consent was not necessary for this work. “We confirm that we have read the Journal's position on issues involved in ethical publication and affirm that this work is consistent with those guidelines.”

## Financial Disclosures and Conflicts of Interest

Author disclosures are available in the Supporting Information.

## Supporting information


**Data S1.** Coi_disclosure_Aquino

## Data Availability

Data sharing not applicable to this article as no datasets were generated or analysed during the current study.
